# Evaluation of inflammatory markers in major psychiatric disorders: challenges and perspectives

**DOI:** 10.47626/2237-6089-2023-0677

**Published:** 2023-10-31

**Authors:** Bianca Wollenhaupt-Aguiar

**Affiliations:** 1 Centre for Clinical Neurosciences Department of Psychiatry and Behavioural Neurosciences McMaster University Canada Centre for Clinical Neurosciences, Department of Psychiatry and Behavioural Neurosciences, McMaster University, Canada.

In the original study by Valiati et al.,^1^ entitled “Inflammation and damage-associated molecular patterns in major psychiatric disorders,” the authors evaluated the peripheral levels of inflammatory markers, assessed by the Damage-Associated Molecular Patterns (DAMPs), heat shock proteins (HSPs) HSP70, HSP60, and S100 calcium-binding protein B (S100B) in a sample of participants with psychiatric disorders, compared to a control (CT) group. DAMPs are intracellular molecules released from the cell to the extracellular environment in response to cellular stress, or death and can activate the immune system.^1^ The authors found that individuals with major depressive disorder (MDD) present higher levels of HSP70, compared to CT and schizophrenia (SCZ) groups. The authors also found that the use of lithium and clozapine was associated with reduced levels of HSP70 in individuals with bipolar disorder (BD) and SCZ, respectively.

This study is very interesting to the field as the authors analyzed a sample of individuals with various mental health conditions, including MDD, BD type I and II, SCZ, and generalized anxiety disorder (GAD). Previous studies have highlighted the relevance of studying biological changes through a transdiagnostic approach.^2^ Changes in biological markers such as inflammatory markers and neurotrophic factors have been extensively reported in major psychiatric disorders but often are assessed in each psychiatric disorder independently^3,4^ rather than through a transdiagnostic approach. Considering that the biological changes may be associated with the pathophysiology of these disorders, studies like the one conducted by Valiati et al. have significant implications to enhance our understanding of the biological basis across major psychiatric disorders.

Another interesting finding from the Valiati et al. study is the fact that around 64% of their total sample exhibited inflammation levels below the assay’s threshold. This is a common issue in psychiatric studies, although not consistently reported. There could be several reasons for this, including substantial heterogeneity among the psychiatric population and low-sensitivity assays. To address the later challenge, high-sensitivity (HS) assays have been developed. However, even so, detection can still pose a challenge for researchers. In addition, different techniques can be used to evaluate inflammatory markers such as enzyme-linked immunosorbent assay (ELISA), cytometric bead array (CBA), and Luminex. In this context, a recent study evaluated the sensitivity of these techniques and others for assessment of inflammatory markers.^5^ The study explores the differences in the most used assays and provides a comprehensive analysis for the selection of these techniques. Nevertheless, new technologies are needed to improve the detection limit in samples with lower concentrations as seen in psychiatric populations. Interestingly, the newest technologies like the single-molecule array (Simoa) have been developed to provide ultrasensitive biomarker detection at femtomolar concentrations and may be highly advantageous in these cases (https://www.quanterix.com/simoa-technology/). Such advances in biological technologies can aid in improving sample detection allowing the assessment of larger sample sizes which may increase the statistical power of the studies, and ultimately, lead to a more comprehensive analysis of the biological underpinnings of major psychiatric disorders.
